# Differential Somatic Cell Count: Value for Udder Health Management

**DOI:** 10.3389/fvets.2020.609055

**Published:** 2020-12-23

**Authors:** Tariq Halasa, Carsten Kirkeby

**Affiliations:** Section for Animal Welfare and Disease Control, Faculty of Medical and Health Sciences, Institute of Veterinary and Animal Sciences, University of Copenhagen, Frederiksberg, Denmark

**Keywords:** management, cow, dairy, mastitis, differential somatic cell count

## Abstract

Intramammary infection (IMI) can cause mastitis, which is one of the costliest and most prevalent diseases in dairy cattle herds. Somatic cell count (SCC) is a well-established parameter to indicate IMI, and it represents the total count of immune cells in the milk. The differential somatic cell count (DSCC) has also long been suggested to indicate IMI, but no machine was available until recently to provide this parameter automatically. Two new machines have recently been introduced to measure the milk DSCC as an additional indicator of IMI. Here we provide insights about the DSCC measured by these two machines and the value it may provide for udder health management, based on the available literature. We also provide perspectives for future research to investigate potential value in using the DSCC to improve udder health.

## Introduction

Mastitis is the inflammation of the mammary gland. It causes welfare and economic damage for the dairy industry worldwide (1). During the past decades, large efforts have been invested to monitor, prevent and control mastitis in dairy cattle herds (2). Several indicators have been proposed to monitor the occurrence of mastitis in dairy cows on a routine basis, including the widely used individual cow somatic cell count (SCC), which represents the total number of immune cells in the milk (3). Several studies have additionally suggested the use of differential somatic cell count (DSCC), which distinguishes between the different immune cells in the milk [see Table 1]. Until recently, the use of DSCC as an indicator of IMI was limited to research studies due to the unavailability of machinery to produce this parameter on a large scale. However, a high throughput machinery for regular Dairy Improvement Health testing (15) and an on-farm machine (16) have recently become available, allowing large scale measurement of DSCC. This warrants the investigation of the usefulness of this parameter for udder health management in dairy cattle herds. Missing information can thereafter be obtained in future research.

**Table 1:** Proportion of polymorphonuclear cells (PMN) lymphocytes (Lym) and macrophages (Mac) observed in different studies, under different definitions of udder health statuses and using different methods of determination of these proportions.

**References**	**Definition**	**Method**	**PMN**	**Lym**	**Mac**
(4)	Healthy: no pathogen and SCC <10,000 cells/ml	Cytology—Microscopy	10	67	23
	1 day post-inoculation with *Staphylococcus aureus*	Cytology—Microscopy	51	28	21
	4–8 days post-inoculation with *Staphylococcus aureus*	Cytology—Microscopy	39	38	24
	9–14 days post-inoculation with *Staphylococcus aureus*	Cytology—Microscopy	32	43	26
	Healthy: no pathogen and SCC <10,000 cells/ml	Flow cytometry	18	72	10
	1 day post-inoculation with *Staphylococcus aureus*	Flow cytometry	61	18	20
	4–8 days post-inoculation with Staphylococcus aureus	Flow cytometry	46	25	29
	9–14 days post-inoculation with *Staphylococcus aureus*	Flow cytometry	31	34	34
(5)^a^	Healthy: SCC <100,000 cells/ml	Flow cytometry	28	11	13
	Acute *E. coli*	Flow cytometry	38	1	4
	Acute *Staphylococcus aureus*	Flow cytometry	36	5	11
	Chronic *Staphylococcus aureus*	Flow cytometry	42	37	17
	Chronic non-aureus Staphylococci	Flow cytometry	49	18	13
	Chronic *Streptococcus dysgalactiae*	Flow cytometry	73	8	5
(6)	SCC <50,000 cells/ml	Flow cytometry	31	26	43
	SCC between 50,000 and 100,000 cells/ml	Flow cytometry	51	14	35
	SCC between 100,000 and 200,000 cells/ml	Flow cytometry	59	12	29
	SCC between 200,000 and 400,000 cells/ml	Flow cytometry	64	11	25
	SCC >400,000 cells/ml	Flow cytometry	67	9	24
(7)	SCC <6,250 cells/ml	Flow cytometry	15	63	22
	SCC between 6,250 and 25,000 cells/ml	Flow cytometry	17	59	24
	SCC between 25,000 and 100,000 cells/ml	Flow cytometry	23	50	27
	SCC >100,000 cells/ml	Flow cytometry	60	19	21
(8)	SCC <6,250 cells/ml	Cytology—Microscopy	12	56	32
	SCC between 6,250 and 25,000 cells/ml	Cytology—Microscopy	12	49	38
	SCC between 25,000 and 100,000 cells/ml	Cytology—Microscopy	52	31	17
	SCC >100,000 cells/ml	Cytology—Microscopy	78	12	10
(9)	Healthy: No pathogen and SCC <100,000 cells/ml	Cytology—Microscopy	43	34	23
	Culture positive and SCC <100,000 cells/ml	Cytology—Microscopy	56	23	21
	Culture negative and SCC >100,000 cells/ml	Cytology—Microscopy	63	16	21
	Culture positive and SCC >100,000 cells/ml	Cytology—Microscopy	68	12	20
(10)^a^	Healthy: no pathogen and SCC <200,000 cells/ml	Flow cytometry	28	58	9
	Moderate mastitis but SCC <100,000 cells/ml	Cytology—Microscopy	47	20	33
	Moderate mastitis and SCC between 100,000 and 400,000 cells/ml	Cytology—Microscopy	59	10	31
	Severe mastitis but SCC <100,000 cells/ml	Cytology—Microscopy	59	18	23
	Severe mastitis and SCC between 100,000 and 400,000 cells/ml	Cytology—Microscopy	71	9	20
	Severe mastitis and SCC >400,000 cells/ml	Cytology—Microscopy	82	5	13
(11)	Healthy: low SCC and no pathogen	Flow cytometry	43	30	27
(12)	Healthy: SCC <100,000 cells/ml	Flow cytometry	32	16	52
	SCC >800,000 cells/ml	Flow cytometry	49	18	33
(13)	Healthy: no pathogen and SCC <100,000 cells/ml	Flow cytometry	20	8	72
	Infused with endotoxin	Flow cytometry	78	2	19
(14)	Healthy: no pathogen and SCC <100,000 cells/ml	Cytology—Microscopy	28	25	47

a *Leucocyte proportions do not necessarily add to 1 because of measuring other cells in the original studies. The numbers were obtained from (6)*.

We here provide insights into the DSCC based on available literature and the potential value it may hold for udder health management. We also provide perspectives for future research to investigate the potential value of using the DSCC in dairy herds.

## Function and Dynamics of Immune Cells Measured in the Milk

The somatic cell count represent the immune cells in the milk, which are mainly lymphocytes, polymorphonuclear neutrophils (PMN) and macrophages (15). The task of the lymphocytes is to regulate the initiation and suppression of the immune response, while the macrophages ingest bacteria and cellular debris (15, 17). In addition, macrophages recognize invading pathogens and trigger an immune reaction, by the rapid recruitment of PMN (15, 18, 19). The task of PMN is to attack the invading pathogen and defend the mammary glands at the start of an acute inflammatory reaction (15, 19).

As immune cells have different functions, their distributions in normal and mastitic milk differs (20). In milk samples obtained from healthy udders, macrophages and lymphocytes dominate the total cells, while PMNs dominate the cell count in milk obtained from infected mammary glands or milk with high SCC (see Table 1).

The proportions of these different leucocytes change following infection. Once the mammary glands are infected, a surge in PMN can be observed [Rivas et al. (4) in Table 1] triggered by the resident cells (lymphocytes, macrophages, and epithelial cells) (21). A few days later, a reduction in the proportion of PMN can be observed, while macrophages eliminate bacteria and debris (4). Sordillo et al. (17) indicated that the severity and duration of a mastitis case is highly related to the promptness of the leucocytes migratory response and the bactericidal activities of the immune cells at the site of infection. If the cells move fast from the blood to the mammary gland and clear the bacteria, cell recruitments ceases. Nevertheless, if the bacteria are capable of surviving the immune reactions, the inflammation continues and may become severe, leading to damage to the mammary gland tissue, which causes production losses (17). The reduction of the proportion of PMN following infection indicates the end of the acute stage, which may result in complete bacterial elimination, in which SCC returns to healthy levels (17). An acute stage maybe followed by a chronic stage, if bacterial elimination is not complete. During this stage, the immune system continues to respond to the presence of bacteria, which is characterized by elevated proportions of PMN [see Leitner et al. (5) in Table 1] and SCC (21). The SCC during the chronic stage may fluctuate, but it will generally remain elevated (21).

## The Relationship between DSCC and SCC

As indicated above, infection of mammary glands results in an increase in SCC and DSCC, when the number and relative proportion of PMN increase (Table 1). Thus, a high correlation between SCC and DSCC is expected, as also shown earlier (6, 22). However, studies have shown that elevations in PMN can be observed at low SCC levels, indicating that active bacterial eliminations without noticeable surges in SCC may take place [see (7–9) in Table 1]. This indicates that more information about the dynamics of udder health on cow level can be obtained by monitoring the DSCC.

Traditionally, the proportions of the leucocytes in milk are measured by either counting cells under a microscope following cell isolation and staining, or using a flow cytometer [see details about both procedures in Rives et al. (4)]. Microscopic examination is perhaps the standard method, as the margins of error for identification of cells are low when carried out by trained persons. Nevertheless, it is cumbersome and can therefore only be used as a tool for research. Rives et al. (4) compared both methods and concluded that flow cytometry is a valid diagnostic approach that provides results comparable to those obtained from manual cytology. Koess and Hamman (6) concluded that flow cytometry could be used reliably to differentiate milk leucocytes and to determine the percentages of the different cell types. These earlier studies paved the way for the development of technology and machinery that utilizes flow cytometry to produce the DSCC in milk.

To our knowledge, two instruments are currently able to measure DSCC in milk samples, automatically. The first machine is produced by FOSS Analytical A/S (Hillerød, Denmark), and is a laboratory based machine that allows high throughput measurement of FOSS DSCC (referred to as F-DSCC throughout the manuscript to distinguish it from the regular DSCC, where the proportions of the different leucocytes are reported) from milk samples using flow cytometry (15). The F-DSCC represent the proportion of PMN and lymphocytes compared to the total number of PMN, macrophages and lymphocytes (15). The second machine (namely QScout) is produced by Advanced Animal Diagnostics (USA), and can be used for on-farm diagnostics. This machine measures the absolute values and proportions of the leucocytes (neutrophils, macrophages, and lymphocytes) in the milk using fluorescent microscopy (16). We refer to the outcome of this machine throughout the paper as Milk Leucocytes differentials (MLD) as presented earlier (23). It is important to point out that limited data is available based in this machine in the literature, perhaps because this machine has only recently been introduced.

## Factors Affecting DSCC

SCC is affected by cow factors such as days in milk (DIM) and lactation number [e.g., Græsbøll et al. (24)]. Dosogne et al. (10) showed that lymphocytes decreased over DIM, while macrophages and PMN increased. On the other hand, Pilla et al. (9) found that only macrophages were significantly influenced by DIM, without finding a significant effect of lactation number or quarter position. However, the authors showed that the percentage of individual cell populations was significantly affected by the tested herd. Furthermore, the herd also affected the ratio of the different cell populations. This could be driven by the differences in the distributions of IMI causing pathogens and levels of IMI between the herds. It could also be attributed to genetic differences (25). Another study showed no significant effect of parity and quarter position on the ratio of the different populations of cells (11).

Kirkeby et al. (26) found a significant association between F-DSCC and both DIM and lactation number. F-DSCC generally decreased over the course of the lactation, and the relationship between F-DSCC and lactation number was found ambiguous (26). Schwarz et al. (27) found no significant effect of lactation number on F-DSCC, while in a more recent study Schwarz et al. (28) reported a significantly higher F-DSCC for cows with lactation number >4 compared to younger cows. In the latter study, no significant association between F-DSCC and DIM was found. In addition, the authors found no significant effect of milk weight (kg milk produced) on F-DSCC (28). In another recent study, the authors found that parity and DIM were significantly associated with F-DSCC (29). In addition, the effect of cow was particularly high (29), indicating that a large part of the variability is attributed to differences between cows. Using the MLD, Lozado-Soto et al. (30) found significant effects of breed, lactation, sampling day, sampling time and quarter position on the MLD measurements. By relating these factors to the MLD measurements, the authors argued that our understanding of the somatic cell count recruitment could be improved, though more studies are needed to validate the findings (30).

These results show that the relationship between DSCC and the key herd- and cow factors is not yet fully resolved, pointing out the need for further studies.

## DSCC in Healthy and IMI Quarters

Only four studies investigated the level of F-DSCC in healthy (no pathogen growth) and IMI quarters (Table 2). In healthy quarters (no pathogen was isolated), the F-DSCC varied between 30 and 78%, while F-DSCC varied between 35 and 96% for IMI quarters.

**Table 2:** Values of differential somatic cell count in healthy and intramammary infected quarters calculated using the DSCC machine produced by FOSS analytical A/S (F-DSCC).

**References**	**Udder health status**	**Value of DSCC**
(26)	Uninfected quarters—Herd 1	Mean = 65%
	Infected with minor pathogen—Herd 1	Mean = 68%
	Infected with major pathogen—Herd 1	Mean = 71%
	Infected with other pathogen—Herd 1	Mean = 73%
	Uninfected quarters—Herd 2	Mean = 68%
	Infected with minor pathogen—Herd 2	Mean = 73%
	Infected with major pathogen—Herd 2	Mean = 74%
	Infected with other pathogen—Herd 2	Mean = 74%
(27)	Uninfected quarters	Mean = 78%, std = 2%^a^
	Infected with minor pathogen	Mean = 88%, std = 2%
	Infected with major pathogen	Mean = 90%, std = 2%
(28)	Uninfected quarters	Mean = 30%, std = 3%^b^
	Infected with minor pathogen	Mean = 35%, std = 3.3%
	Infected with major pathogen	Mean = 68%, std = 3.5%
	Infected with other pathogen	Mean = 34%, std = 3.7%
(31)^c^	Uninfected quarters—Group A	52–60%
	Infected quarters with lipopolysaccharides—Before infusion (healthy status)	54–61%
	Infected quarters with lipopolysaccharides—after infusion (infected)	69–94%
	Infected quarters with lipoteichoic acid—before infusion (healthy status)	45–47%
	Infected quarters with lipoteichoic acid—after infusion (infected)	54–96%

a *Statistically different than infected quarters*.

b *Statistically different than infected quarters with major pathogens*.

c *Experimental study*.

The MLD machine provides absolute and proportions of the milk leucocytes. Thus, these outcomes can be directly compared to leucocytes absolute values and proportions of milk obtained from healthy quarters (e.g., see Table 1) to indicate subclinical mastitis.

## DSCC as Indicator for IMI

The relationship between DSCC and IMI causative pathogens has been studied also. Leitner et al. (5) found that the distributions of leucocytes in quarters infected with *Streptococcus dysgalactiae* did not differ from that in quarters that had acute IMI caused by *Staphylococcus aureus* or *Escherichia coli*. Furthermore, the authors found no significant difference in PMN proportions between quarters that had chronic IMI caused by *Staphylococcus aureus* or non-aureus staphylococci (NAS) compared to healthy quarters. Wall et al. (31) studied the reaction of F-DSCC to IMI induced by lipopolysaccharide (LPS) or lipoteichoic acid (LTA), which resembles an *Escherichia coli* and *Staphylococcus aureus* IMI, respectively. The authors found a sharp increase in the F-DSCC a few hours after treatment with LPS or LTA from, 60 to 80%, indicating a shift in cell populations toward PMN. This indicates that the F-DSCC is capable of representing the change in DSCC following IMI.

Kirkeby et al. (26) investigated the association between F-DSCC and healthy quarters or quarters with IMI caused by major, minor and other IMI causing pathogens. The authors found a significant difference in F-DSCC between healthy and IMI quarters, also when SCC was already taken into account. However, this effect was influenced by a herd effect and the IMI causative pathogen group. Similarly, Schwarz et al. (27) studied the efficacy of F-DSCC to indicate IMI at dry off, and found that quarters with IMI had significantly higher F-DSCC than healthy quarters. Nevertheless, no significant difference in F-DSCC was observed between quarters that had IMI caused by major and minor IMI causing pathogens. In a subsequent study, Schwarz et al. (28) found that F-DSCC was significantly higher in quarters with IMI caused by major pathogens compared to healthy quarters and quarters with IMI caused by minor pathogens. In addition, the authors found that the F-DSCC response differed for different NAS pathogens (28). Another recent study investigated the association between F-DSCC and the IMI status of cows with SCC <50,000 cells/ml (32). For these cows, the authors found no significant association between F-DSCC and the IMI status.

Schwarz et al. (28) calculated the sensitivity and specificity of F-DSCC to indicate IMI. Using an F-DSCC cut-off value (threshold value to indicate IMI) of 60%, the sensitivity and specificity were estimated to 87.42 and 67.26%, respectively (28). Increasing the cut-off increased the sensitivity, but it reduced the specificity (28). The sensitivity could be further improved by combining the selection based on F-DSCC with SCC, as initially intended for the F-DSCC use (15), but this also reduced the specificity (28). Similar findings were reported by Schwarz et al. (27).

The sensitivity and specificity of the MLD using single or duplicate milk samples were measured by Godden et al. (23). The sensitivity varied between 25 and 86%, while the specificity varied between 32 and 95%, depending on the setting of the machine to optimize sensitivity or specificity. Nevertheless, the authors recommended the use of a cow sample (pooled milk sample from the four quarters of the udder) as it improved the sensitivity (23). Comparable values of the sensitivity and specificity were found by Lozado-Soto et al. (30).

Using a bioeconomic simulation model, Gussmann et al. (33) estimated the added value of using F-DSCC to indicate subclinical IMI when SCC is already known. In the simulated scenarios, the farmer selected cows based on F-DSCC and/or SCC for bacterial culturing and subsequent treatment and culling decisions in case of subclinical IMI. The authors found that using F-DSCC additionally to SCC for the selection would not affect the economic outcomes or the IMI status in the herd, but it would result in lower antibiotic usage.

## DSCC and Milk Production

One study investigated the association between milk production and different udder health groups based on the combination of F-DSCC and SCC (34). The authors classified cows based on their SCC and F-DSCC status into low F-DSCC and SCC (Group A), high F-DSCC and low SCC (Group B), high F-DSCC and SCC (Group C) and low F-DSCC and high SCC (Group D). They found that cows in groups B, C, and D produced 0.9–2.4%, 6–9.8%, and 17.5–38.5% less milk than cows in group A, respectively. The same approach was used by Bobbo et al. (35) to study the association between milk yield and composition and SCC combined with F-DSCC. The authors found that cows in group B had slightly higher milk production than those in group A, while cows in groups C and D had lower milk production than those in groups A and B. The picture was opposite for fat and protein production, perhaps due to a concentration effect as a result of the reduced milk production (35). In another recent study, Zecconi et al. (32) found no significant association between milk production and F-DSCC for cows with SCC <50,000 cells/ml.

## Strengths and Limitations of DSCC for Application to the Field: Perspectives

As presented above and in Table 1, numerous studies have been conducted to investigate the ability of DSCC to indicate IMI. These studies focused on the theoretical understanding of the behavior of the different leucocytes in milk as a response to IMI, in order to establish the usefulness of differentiating these cells to indicate IMI. The work has shown evidence that differentiating milk leucocytes can indicate IMI.

The F-DSCC machine provides a measure of the DSCC in one value. It can be used for high throughput, and hence it provides the possibility to monitor cows regularly, which makes it quite practical and attractive for register-based applied research. The MLD machine on the other hand provides several measurements, which may give more insight into the reactions of the immune system toward infection. The MLD machine is not designed to provide high throughput analyses, which may limit its use as a tool to regularly monitor cows in dairy herds. However, it can be used on farm and hence measurements can be obtained for specific cows when desired.

The F-DSCC parameter is capable of indicating IMI during lactation and at dry off (26, 28) and may react differently between IMI with major and minor pathogens (26). Nevertheless, comparing values from four different studies (Table 2), F-DSCC may vary largely in both healthy and IMI quarters. This and the fact that several factors may affect F-DSCC, makes it difficult to set simple thresholds to discriminate healthy from IMI quarters using F-DSCC based on the currently available litterature. Therefore, further research is needed to establish IMI detection using F-DSCC both with and without SCC, and the optimal cut-off values should be determined, to facilitate the use of these parameters for udder health management. In addition, influential cow- and herd factors should also be considered when these cut-off values are proposed, as they may aid in providing cow- and herd-specific decision support to manage udder health. Gussmann et al. (33) used a threshold of 62% to identify cows with subclinical mastitis for treatment and/or culling decisions and showed that using F-DSCC combined with SCC (threshold of 200,000 cells/ml) can improve the selection of the cows and result in reduced antibiotics usage, while improving udder health. Further work investigating the effectiveness of using these parameters to select cows for dry cow therapy is needed. This may aid in further reduction of antibiotics usage in dairy cattle herds. In addition, the value of F-DSCC for breeding should be investigated thoroughly, as recent work showed that it may have the potential to improve udder health (25). Nevertheless, the authors recommended further work to quantify the weight that F-DSCC should receive in breeding programs to improve udder health (25).

The MLD machine provides proportions and absolute values of the milk leucocytes (16). This can provide insight into the immune system reactions toward IMI causing pathogens (30), and hence improve our understanding of how to tackle IMI caused by the different pathogens; e.g., when treatment is needed. Nevertheless, a better understanding of these relationships based on these measurements is necessary before recommendations can be made. In addition, production effects in relation to these measurements should be established, to allow conducting economic assessments based on these measurements. This will also facilitate their use from a practical standpoint. Thus, more work is needed to inform how the MLD measurements should be used to manage udder health and what value this may bring.

Finally, it is important that future research focuses on conducting field studies comparing management practices with and without using the DSCC, in order to validate the usefulness of this parameter under field conditions and allow translating research findings into the field. This would aid in the establishment of general guidelines for the use of DSCC to improve udder health in dairy cattle herds.

## Data Availability Statement

The original contributions presented in the study are included in the article/supplementary material, further inquiries can be directed to the corresponding author.

## Author Contributions

All authors listed have made a substantial, direct and intellectual contribution to the work, and approved it for publication.

## Conflict of Interest

The work in this paper was part of a large research project, in which FOSS Analytical A/S was a partner. The authors declare that the research was conducted in the absence of any commercial or financial relationships that could be construed as a potential conflict of interest.
